# Increasing health facility childbirth in Ghana: the role of network and community norms

**DOI:** 10.1186/s12884-023-05513-9

**Published:** 2023-04-19

**Authors:** Leslie E. Cofie, Clare Barrington, Kersten Cope, Catherine E. LePrevost, Kavita Singh

**Affiliations:** 1 Department of Health Education and Promotion, East Carolina University, 3104 Belk Building, Greenville, NC 27858 USA; 2 Department of Health Behavior, Gillings School of Global Public Health, University of North Carolina, 302 Rosenau Hall, Chapel Hill, NC CB #744027599-7440 USA; 3 Carolina Population Center, University of North Carolina, CB#81200, Chapel Hill, NC 27599-7440 USA; 4 University of South Carolina, Health Promotion, Education, and Behavior, 915 Greene Street, Columbia, SC 29208 USA; 5 Department of Applied Ecology, North Carolina State University, 237 David Clark Labs, Raleigh, NC 27695 USA; 6 Department of Maternal and Child Health, Gillings School of Global Public Health, University of North Carolina, Rosenau HallChapel Hill, NC CB #744527599-7445 USA

**Keywords:** Maternal health, Social norms, Community norms, Network norms, Health facility birth

## Abstract

**Background:**

Reducing pregnancy-related deaths in Sub-Saharan Africa through increases in health facility births may be achieved by promoting community norms and network norms favoring health facility births. However, the process of how both norms shift attitudes and actions towards facility delivery is little studied. We examined the association of network and community norms with facility birth, following a quality improvement intervention to improve facility births in Ghana.

**Methods:**

A 2015 mixed methods evaluation of a Maternal and Newborn Health Referral (MNHR) project in Ghana included a cross-sectional survey of women (*N* = 508), aged 15–49 years; in-depth interviews (IDIs) with mothers (*n* = 40), husbands (*n* = 20) and healthcare improvement collaborative leaders (*n* = 8); and focus group discussions (FGDs) with mothers-in-law (*n* = 4) and collaborative members (*n* = 7). Multivariable logistic regression was used to examine the association of network and community norms with facility birth. Thematic analysis of the qualitative data was conducted to explain this relationship.

**Results:**

The network norm of perceived family approval of facility delivery (AOR: 5.54, CI: 1.65–18.57) and the community norm of perceived number of women in the community that deliver in a facility (AOR: 3.00, CI: 1.66–5.43) were independently associated with facility delivery. In qualitative IDIs and FGDs both norms were also collectively perceived as influencing facility delivery. However, network norms were more influential in women’s utilization of facility-based pregnancy-related care. Healthcare improvement collaboratives were important in swaying both network and community norms toward facility-based delivery by offering pregnancy-related health information, antenatal care, and support for facility delivery.

**Conclusion:**

Quality improvement initiatives impact both community and network norms. To be most impactful in advancing facility-based pregnancy-related care, these initiatives should focus on highlighting the shifting trend toward facility delivery in rural communicates and promoting support for facility delivery among women’s personal networks.

## Plain English summary

Social norms are shared values, beliefs, and attitudes within a group, and they may impact individuals’ behaviors, including their health behaviors. We studied the relationship between norms and women’s decisions to deliver their child in a facility in rural Ghana. We surveyed 508 women and interviewed 79 participants including mothers, their husbands, mothers-in-law, community leaders and health care providers. We learned that women’s beliefs that their families approved of facility delivery and that other women in their community delivered in a facility were important in their decision making. Community teams made up of community leaders and health care professionals had a positive impact on women’s beliefs and their decisions to deliver in a facility. Community-based efforts to increase facility delivery should help women’s support systems, made up of family members such as husbands and mothers-in-law, understand their important role in encouraging women’s access and use of health facilities.

## Background

Sub-Saharan Africa accounts for approximately two-thirds of the global maternal deaths [1]. Specifically, in Ghana the maternal mortality ratio remains high at 310 deaths per 100,00 live births [2]. As many maternal deaths can be prevented through pregnant women’s use of health facility birth with the assistance of a skilled birth attendant [3, 4], efforts have been made in the past decade to increase facility-based childbirth in many sub-Saharan African countries [2]. In Ghana, health facility births increased from 55% in 2008 to 79% in 2017. This increase can be attributed to nationwide initiatives to implement intervention strategies to improve maternal health services use, as well as the Ghana health ministry’s implementation of free national health insurance and maternal delivery services [5]. Facility births in rural areas of Ghana, where more than half of the nation’s population reside, are significantly lower (69%) than the national average [2]. Reasons for this include limited access to health facilities, cost and time associated with utilizing the facilities, and social factors such as social norms related to childbirth practices [6].

Research to improve maternal health services utilization has in part focused on social norms as important determinants of place of childbirth. Social norms refer to shared values, beliefs, or attitudes of a group that impact perceptions and behaviors of the group members [7]. These norms may be sanctioned by influential individuals within a community or community members [8]. Community norms favoring facility birth have been positively associated with an increased rate of childbirth in health facilities [9–11]. Normative beliefs about the importance and quality of health facility birth within a community may be shaped by health outreach and intervention strategies and, in turn, increase the likelihood of health facility births among women [11, 12]. In Ghana, studies have pointed to a shifting trend toward community acceptance of health facility birth, due to changing perceptions about the benefits and quality of care in these facilities [13].

Social norms may also be sanctioned by members of one’s own personal network such as relatives and friends. There is limited evidence related to the relationship between network norms and health facility birth [14]. Our recent examination of the role of personal network characteristics in women’s place of childbirth revealed that women with greater numbers of close relatives that approve of facility birth, and knowledge of more women in their network that received pregnancy-related care in a facility, are more likely to give birth in a health facility [15]. Less is known about the interactive impact of network and community norms on facility birth.

Social network theories indicate the importance of connections and norms to health behaviors [7, 16, 17]. For example, network and community norms have each been individually examined as influential determinants of health decisions about women’s pregnancy-related care continuum [10–12, 18]. Previous work has linked community perceptions about the quality of formal health systems with facility delivery [12], and across six African countries, community-level norms – e.g., percentage of women in the community that delivered in a health facility, husbands in the community that approve of family planning – were associated with women’s decision to deliver at a health facility [19]. Specifically in Ghana, Speizer and colleagues (2014) found a significant association between norms about facility births and women’s use of facility delivery, as well as a significant interaction between these norms and women’s decision-making autonomy. Whereas research is limited on network norms and facility births, recent evidence from Ghana suggests that normative support for facility birth from network members was associated with facility delivery [18]. Both norms can inform health intervention programs and strategies to further improve uptake in health facility births. However, it is unknown whether network or community norms are more salient than the other, or if both norms have a cumulative effect on health facility birth.

The purpose of the present study is to examine the association of network and community norms with facility birth, following a quality improvement intervention to improve facility births in the Northern and Central regions of Ghana; both regions are representative of the populations impacted by their birth delivery preference. We hypothesized that network and community norms will each be positively associated with, and have an interactive effect on, facility birth.

## Methods

### Study design

A Quality Improvement Maternal and Newborn Referral project in Ghana was implemented to improve the referral process for pregnant women and sick newborns in need of comprehensive medical care [20]. We have previously described the study setting [15]. In this study, we examined data from the project’s end-line assessment that was conducted in 2015. We used a mixed methods convergent design to separately collect and analyze quantitative and qualitative data within the same period; and the qualitative findings enabled the interpretation of the quantitative findings [21]. As women’s perceptions of their social networks are well correlated with measured attributes of their network members [22], we used egocentric network measures to assess rural Ghanaian women’s social networks [23].

### Study sampling and participants

The quantitative study was a cross-sectional community-based study using a 3 by N cluster sample design, as detailed elsewhere [18]. Within each of 3 designated districts in the Northern Region (NR), 30 communities were randomly selected to be either an intervention or a control community; the same approach was used in the Central Region (CR). A total of 1,260 women including those with recent birth and those of reproductive age were interviewed. The present study focused on women with pregnancy in the past 3 years (*N* = 818), and the analytic sample was 508 after excluding women who had missing information on all key variables of interest.

Using a purposive sampling approach, the qualitative study as detailed elsewhere included both in-depth interviews (IDIs) with women and focus-group discussions (FGDs) [18]. We recruited a subsample of the survey participants (*n* = 40) for the IDIs. A sample of these women’s husbands (*n* = 20) was interviewed. FGDs were conducted with mothers-in-law (MILs, *n* = 4) selected from a community in the district where the IDIs were conducted. Additional IDIs were conducted with leaders of healthcare improvement collaboratives (*n* = 8) made up of health facility heads. FGDs (*n* = 7) were also conducted with members of healthcare improvement collaboratives consisting of both health facility workers and community leaders involved in the process of improving the referral process.

### Data collection

For the quantitative study, we developed and administered household surveys to the women. Interview guides and focus guides were also developed to collect qualitative data from women, husbands, MILs, and members of the healthcare improvement collaboratives. The IDIs (~ 60 min) and FGDs (~ 90 min) were audio recorded in locations selected to protect confidentiality that could be indoors or outdoors or in a private community space and were later transcribed into English. Male and female research assistants, who received training and were experienced in data collection in the local languages of the study communities (Twi and Fanti in the CR and Dagbani and Lekpepkel in the NR), conducted household surveys, IDIs, and FGDs in the local languages. This study was exempted from ethics review by University of North Carolina (UNC)–Chapel Hill’s Internal Review Board, as it was considered a program evaluation. All participants verbally consented before participating in the study.

### Quantitative measures

The outcome measure, health facility birth, was based on whether women participants delivered at a health facility during their most recent pregnancy. The response was dichotomized, yes or no.

Key independent measures included network and community norms adopted from existing measures and review of the network literature [8, 24–27]. Network norms, *family and friend approval of facility birth*, were measured as: “how much do your (1) close relatives, and (2) friends, you described in the previous section approve of or encourage the use of health facilities for care during pregnancy and childbirth?” Response options were recoded into higher approval (strongly approve or approve) and lower approval (somewhat approve, do not approve, or not applicable). Additionally, *network sought facility care*, was derived from the question: “How many of the women you know of (e.g., relatives, friends, and acquaintances) have gone to the health facility for their pregnancy related care?” The response categories were recoded as greater number (most or many), some, and fewer number (few or none). Community norm measures were perceived number of *women that deliver in facility*, *men that support facility birth*, and *MILs’ attitudes toward facility delivery*. The variables were based on the following successive questions: 1. “How many women do you think in your community deliver their baby in a health facility?” 2. “In your opinion, what percentage of men in your community is supportive of facility delivery?” 3. “In your opinion, what percentage of mothers-in-law in your community is supportive of facility delivery?” Response options for each question were recoded into majority (all/most) and minority (some/few/none).

Control measures, associated with use of health facility delivery, included maternal age, education, employment, household wealth, religion, marital status, ethnicity, parity, region, and decision-making autonomy [28]. We created the wealth variable based on a similar approach used in previous studies [11, 20]. We used three household characteristics – type of fuel used, type of toilet, and location of kitchen– to assess wealth. The poorest households were those that (1) use wood for fuel, (2) have a non-improved toilet (definition from the Ghana Demographic Health Survey) and (3) have a kitchen outside the house. We coded households with two out of three of these characteristics as medium wealth, and households with one or none of these characteristics as wealthiest. The autonomy variable was based on the item: “who usually makes decisions about health care for you?” Response options were as follows: respondent alone, partner alone, or others (respondent and husband/partner jointly or network members).

### Qualitative instruments

We developed interview guides for the IDIs and FGDs. These included questions about women’s and their husband’s experiences and perceptions of the role of network and community norms in their pregnancy and childbirth-related care. Husbands were also asked to describe their role as well as their network's involvement in health decisions and support for their wives’ pregnancy and delivery. MILs were asked to describe their role as well as their network's involvement in health decisions and support for women’s pregnancy, and to describe community perceptions about place of childbirth. Leaders and members of healthcare improvement collaboratives also provided information on their work with community members and women’s families to promote facility-based pregnancy care.

### Data analysis

Quantitative analyses were conducted in SAS version 9.34 (SAS Institute, Cary NC). We examined whether network and community norms and control variables were associated with facility birth using chi-square test and t-test. Multivariable logistic regression models were used to test the association between network and community norms with facility births, adjusting for control measures. We also assessed whether the interaction between community and network norms had an effect on facility birth [29]. The regression analyses were two-tailed (p < 0.05) and adjusted for clustered survey design.

Qualitative analysis began with a close reading of all transcripts (by LEC and KC) in Atlas.ti software (version 7.0, Scientific Software Development GmbH, Eden Prairie, MN). Subsequently, the research team, including LEC, KC and CEL, discussed emerging themes from the transcripts, memos, and fieldnotes, which resulted in development of preliminary codebook for thematic analysis. LEC and KC applied the codes to the transcripts and refined the codebook with input from the team [30]. LEC conducted coding checks to ensure that the coded data reflected codes defined in the codebook. Discrepancies identified were discussed and resolved by the research team (LEC, KP, and CEL) to ensure coding accuracy and consistency. The research team analyzed and interpreted the data by reviewing the code outputs and developed code summaries and analytic matrices [31, 32]. This yielded emerging themes capturing study participants’ perceptions of how network members impact women’s pregnancy and birth experiences, as well as community perceptions about norms surrounding pregnancy. The matrices enabled comparison of community and network influences. Also, we identified common themes shared by all participants by comparing the experiences and perceptions of women, husbands, MILs, and members and leaders of the health care improvement collaboratives.

## Results

### Quantitative findings – descriptive characteristics

Over half of all women (53%) were 25–34 years of age (Table 1). More women who had homebirth had no formal education than women who had facility birth (75% vs. 41%, *p* < 0.01). A greater proportion of women who had homebirth than those who had facility birth did unpaid work or were unemployed (83% vs. 58%, *p* < 0.01), were in the poorest household wealth category (73% vs. 49%, *p* < 0.01), were married or living with a partner (96% vs. 88%, *p* < 0.01), had more children (4.1, SD 4 vs. 3, SD 2, *p* < 0.01), and indicated that their husband alone made decisions about their health care (77% vs. 59%, *p* < 0.01).

**Table 1:** Descriptive and network and community norm characteristics of women with most recent childbirth, and by place of birth

Descriptive characteristics	Total sample (*N* = 508)	Homebirth (*n* = 240)	Facility birth (*n* = 268)
	n (%)	n (%)	tn (%)
**Age**			
< 19 years	15 (3.0%)	3 (1.3%)	12 (4.5%)
19 – 24 years	126 (24.8%)	51 (21.3%)	75 (28.0%)
25 – 34 years	271 (53.4%)	138 (57.5%)	133 (49.6%)
35 – 49 years	96 (18.9%)	48 (20.0%)	48 (17.9%)
Education***			
None	288 (56.7%)	179 (74.6%)	109 (40.7%)
Primary	111 (21.9%)	41 (17.1%)	70 (26.1%)
Secondary	109 (21.5%)	20 (8.3%)	89 (33.2%)
Employment***			
Paid	16 (3.2%)	2 (0.8%)	14 (5.2%)
Self employed	137 (27.0%)	38 (15.8%)	99 (36.9%)
Unpaid/unemployed/other	355 (69.9%)	200 (83.3%)	155 (57.8%)
Household wealth***			
Richest	68 (13.4%)	15 (6.3%)	53 (19.8%)
Medium	136 (26.8%)	51 (21.3%)	85 (31.7%)
Poorest	304 (59.8%)	174 (72.5%)	130 (48.5%)
Religion***			
Christian	244 (48.0%)	97 (40.4%)	147 (54.9%)
Moslem	179 (35.2%)	78 (32.5%)	101 (37.7%)
None/traditional/Other	85 (16.7%)	65 (27.1%)	20 (7.5%)
Marital status***			
Married/living together	466 (91.7%)	230 (95.8%)	236 (88.1%)
Not currently in union	42 (8.3%)	10 (4.2%)	32 (11.9%)
Ethnicity***			
Akan	147 (29.0%)	34 (14.2%)	113 (42.2%)
Mole-Dadgbani	164 (32.3%)	74 (30.8%)	90 (33.6%)
Grum	98 (19.3%)	64 (26.7%)	34 (12.7%)
Other	99 (19.5%)	68 (28.3%)	31 (11.6%)
**Parity**			
Mean(SD)	3.42(SD ± 2.2)	4.1 (SD ± 2.4)	2.95 (SD ± 2)
Region***			
Central	164 (32.3%)	42 (17.5%)	122 (45.5%)
Northern	344 (67.7%)	198 (82.5%)	146 (54.5%)
Usually makes decision about your health care***			
Husband alone	459 (67.1%)	236 (77.4%)	223 (58.8%)
Respondent alone	185 (27.1%)	50 (16.4%)	135 (35.6%)
Other	40 (5.9%)	19 (6.2%)	21 (5.5%)
Family approve health facility care***			
Higher approval	460 (90.6%)	198 (82.5%)	262 (97.8%)
Lower approval	48 (9.5%)	42 (17.5%)	6 (2.2%)
Friend approve health facility care**			
Higher approval	473 (93.1%)	215 (89.6%)	258 (96.3%)
Lower approval	35 (6.9%)	25 (10.4%)	10 (3.7%)
Network members known to have facility birth***			
Most	314 (61.8%)	124 (51.7%)	190 (70.9%)
Some	144 (28.4%)	77 (32.1%)	67 (25.0%)
Few	50 (9.8%)	39 (16.3%)	11 (4.1%)
Women in community that have facility birth***			
All/most	307 (60.4%)	101 (42.1%)	206 (76.9%)
Some/none	201 (39.6%)	139 (57.9%)	62 (23.1%)
**Men in community that support facility birth** **			
All/Most	363 (71.5%)	153 (63.8%)	210 (78.4%)
Some/few/none	145 (28.5%)	87 (36.3%)	58 (21.6%)
**MILs in community that support facility birth** ***			
All/Most	351 (69.1%)	142 (59.2%)	209 (78.0%)
Some/none	157 (30.9%)	98 (40.8%)	59 (22.0%)

Sample size is slightly smaller for some variables that had missing data. Significance tests compare homebirth with facility birth; *p ≤ 0.05; **p ≤ 0.01; ***p ≤ 0.001; ****p ≤ 0.0001

In terms of network norms, there was a significant difference between women who had facility and homebirth in their perceptions that close family members (98% vs. 83%, *p* < 0.01) and close friends (96% vs. 90%, *p* < 0.01) had higher approval of facility-based pregnancy and delivery care (Table 1). More women who had facility delivery than homebirth perceived that most women they know have had facility-based pregnancy care (71% vs. 52%, *p* < 0.01). Related to community norms, more women who had facility birth compared with homebirth indicated that most women in their community have facility-based childbirth (77% vs. 42%, *p* < 0.0.01), and men (78% vs. 64%, *p* < 0.0.01) and MILs (78% vs. 59%, *p* < 0.0.01) in their community were supportive of facility birth.

### Qualitative findings – network and community norms about place of childbirth

Overall, 17 out of the 36 women gave birth at a health facility. Interview participants highlighted ways in which both network and community norms played a role in women’s pregnancy and delivery experiences. They provided insights into how each type of norm impacted women’s place of childbirth. In terms of network norms, various study participants described ways in which network members uniquely informed or directly influenced the birth location experiences of mothers. The role of network norms in women’s pregnancy and delivery experiences was mostly expressed by mothers that experienced, or intended to experience, health facility delivery. A notable exception was one mother (NR Farmer, 20 yrs.) who maintained that her sister influenced her decision to have homebirth.

Most mothers remarked that their network members (including family, close friends, and neighbors) had successfully experienced facility births, and thus advised them to utilize facility delivery. For example, a NR mother (unemployed, 20 yrs.) mentioned that her husband and sister influenced her decision to give birth in a health facility. In describing her sister’s role, she explained, “*We were both pregnant, but she gave birth before me at the hospital, so she advised me to deliver at the hospital.”* Several women received this type of advice from various network members. For instance, a CR mother (unemployed, 22 yrs.) mentioned that her sister-in-law, grandmother, and friend influenced her decision to give birth in a health facility through the advice she received.

Interviewer: Why was [your sister-in-law] in support to you giving birth at the hospital?

Respondent: She said giving birth at the hospital is better than home birth so I should go to the hospital.

Interviewer: How about [your grandmother]?… [Friend]?

Respondent: She too the same; she said giving birth at the hospital is better than home birth.

Another CR mother (Trader, 37 yrs.) who received similar advice from her sisters offered insight into the magnitude of influence her family had on her place of birth. She stressed, “*In this house, everybody goes to deliver at the hospital*.” This quote highlights a distinction between network and community expectations. Network members enforced their normative expectations through the various types of support (for example, advice and resources) they offered to women during their pregnancy continuum. In addition to advice from their network, women received resources including money, transportation, food, and help with housework, which enabled them to access and use facility-based antenatal, labor, and delivery care.

Related to community norms, many women and husbands perceived that facility or homebirth utilization among women was based on community expectations or perceptions of where women should give birth. Several women echoed the sentiment of a 23-year-old mother (NR, Tailor): “*In this place if it is not critical, you do not go to the hospital.”* She explained that although women may sometimes go to a health facility for management of a complicated childbirth, it was customary for women to have homebirth. Therefore, she fully anticipated giving birth at home.

Interviewer: Do you know of any woman who gave birth at home and not in the hospital?

Mother: Yes.

Interviewer: Who talked to you about giving birth at home that interested you?

Mother: No one talked to me about home delivery. I was just seeing it myself.

Interviewer: [You had homebirth] because you were seeing them give birth at home?

Mother: Yes.

Women’s adherence to community norms also extended to health facility delivery. Many women and husbands described their interactions with health providers as part of the pregnancy care experience. They considered facility birth an essential part of ensuring the health of both mother and infant. A 37-year-old farmer (NR husband) offered the following clarification.

*The reason [for allowing my wife to go to the health facility] is that I have seen a lot of women in this community delivering there, so if there is problem, they [health providers] will manage it. They are the best people to handle it, if my wife has any complication during delivery.*

### Quantitative findings – relationship between norms and health facility delivery

The relationship between each network and community norm and health facility birth among women was examined controlling for sociodemographic factors including age, education, employment, household wealth, parity, marital status, religion, ethnicity, region, and decision-making autonomy (Table 2, Unadjusted Models). In terms of network norms, women who perceived that their close family had a higher approval of facility-based pregnancy and delivery care had higher odds of having facility birth (OR: 7.21, CI: 2.20–23.70) than those who perceived their family had a lower approval. Respondents perceiving that most women they know have had facility-based pregnancy care had higher odds of having facility birth (OR: 3.05, CI: 1.27–7.34) than those who perceived few women they know had facility care. For community norms, facility birth was associated with women’s perception that most women in their community have had facility birth (OR: 3.64, CI: 2.07–6.41), and that most MILs in their community were supportive of facility birth (OR:2.62 CI: 1.40–4.90).

**Table 2:** Association between network and community norms with health facility birth among women

Norms	**Health facility birth OR (95% CI)**	
	**Unadjusted Models**	**Adjusted Model**
**Family approve health facility care**		
Higher approval	7.21 (2.20–23.70)**	5.54 (1.65–18.57)**
Lower approval	Ref	Ref
**Friends approve health facility care**		
Higher approval	2.0 (0.729–5.46)	–
Lower approval	Ref	–
**Network members known to have facility birth**		
Most	3.05 (1.27–7.34)*	1.27 (0.42–3.85)
Some	2.42 (0.95–6.16)	1.65 (0.51–5.37)
Few	Ref	Ref
**Women in community that have facility birth**		
All/most	3.64 (2.07–6.41)***	3.00 (1.66–5.43)***
Some/none	Ref	Ref
**Men in community that support facility birth**		
All/Most	1.78 (0.98–3.25)	–
Some/few/none	Ref	–
**MILs in community that support facility birth**		
All/Most	2.62 (1.40–4.90)**	1.70 (0.93–3.11)
Some/none	Ref	Ref

Note: Each cell in the unadjusted models column is a separate model. The Adjusted model column adjusted for network and community norms measures that are significantly associated with facility birth. The models controlled for age, education, employment, household wealth, parity, marital status, religion ethnicity, region, and who usually make decision about your healthcare. *p < 0.05; **p < 0.01; *p* < 0.001

Network and community norms had no interaction effect on location of birth, and thus a final model adjusted for these measures, in addition to the sociodemographic controls (Table 2, Adjusted Model). Women’s perception that their close family had a higher approval of facility-based pregnancy and delivery care (OR: 5.54, CI: 1.65–18.57), and that most women in their community have had facility birth (OR: 3.00, CI: 1.66–5.43), remained significantly associated with facility birth; the strength of associations were attenuated.

### Qualitative findings – impact of healthcare improvement collaboratives on norms

Women, husbands, and MILs, as well as members of healthcare improvement collaboratives, indicated that health outreach efforts were important in swaying network and community norms toward facility-based delivery. Most perceived that network norms were more resonant in the normative shift toward facility-based care. For example, collaborative members engaged in promoting health facility care for pregnant women indicated that health outreach efforts were designed to cater to both women and their networks as well as the community at large. Through the collaboratives, health providers offered women pregnancy-related health information, antenatal care, and support for facility delivery. As explained by a health care improvement collaborative member (CR FGD, Participant 11), *[we accomplish our goals] through health education and effective home visiting. Sometimes during the antenatal meetings, we do health talks on how to prevent anemia, and when we go on home visits too, we do the same thing.* Home visits and women’s facility care visits provided an avenue for health providers to directly encourage facility birth among women and their network members.

In targeting various communities, they also visited churches, mosques, and community gatherings such as durbars or festivals. One collaborative member explained that education to promote women’s utilization of facility delivery was accomplished *through going to churches and giving them talks* (NR FGD, Participant 9). Another participant (CR husband) explained: *That they [collaboratives] also have community volunteers; they also gave advice on the food they should be eating, the way they should be living for healthy life and safe delivery*. In addition to using large gatherings as avenues to educate community members, health providers worked with trusted and prominent community leaders to facilitate community acceptance of facility delivery. Notably, the head of the collaborative (CR) described a community leader as an *ambassador* to his community as he encouraged women to utilize facility-based pregnancy delivery.

### Qualitative findings – normative shift towards facility birth

Participants including women, MILs, and collaborative members contextualized women’s experiences by expounding that across many communities there has been a shift from homebirths to facility births. In their view, community norms have begun transitioning from home births to facility delivery due to the increase in education outreach that has enabled women’s networks to facilitate pregnant women’s use of facility-based care, by advising women and providing them with needed resources (e.g., transportation, money, and help with household work). As explained by the head of the collaborative (NR, Community health nurse, 32 yrs.), although initially women and their network facilitated homebirth,

*Their perception about the hospital or formal health care is now good. We have a number of drugs they will give to pregnant women. But when women give birth at home, they cannot be certain of the drugs the birth attendant will give them. They see the medication in the hospital as something better.*

Whereas facility or homebirth for some mothers was based on observing customs in their communities, for most women, network members that are traditionally involved in their pregnancy experiences had a prominent role in decisions about their place of childbirth. Most participants acknowledged that many women’s interactions with their networks (e.g., husbands, MILs, sisters, rivals, and close friends) resulted in use of facility delivery. A collaborative member (NR Farmer, 50 yrs.) identified the shift in husbands’ support from home to facility birth:

*Most men preferred home deliveries to hospital deliveries before Project Five Alive (community outreach). But now that we are educating them, they know that hospital deliveries are safer than home deliveries. Many men are now allowing their wives to deliver at the hospital.*

This quote demonstrates the change in perceptions about facility delivery, and the effectiveness of outreach efforts to reorient network norms toward facility birth. Although it highlights husbands’ roles, participants also mentioned that other network members including in-laws were a critical part of the effort to improve women’s use of facility-based delivery.

## Discussion

We found that network and community norms were independently associated with facility-based delivery among women in rural Ghana. In terms of community norms, women who perceived that other woman in the community have facility birth had a higher odds of facility birth than those who perceived other women did not. The odds ratio for network norms was, however, higher than community norms; women who perceived that their families approved of health facility care had five times higher odds of having facility birth than those who perceived their families did not. The qualitative findings suggest that both network and community norms impacted women’s use of health facility care. Health outreach efforts involving healthcare improvement collaboratives influenced the behaviors of women in accessing and using pregnancy-related care including facility-based delivery. However, in alignment with the quantitative findings, study participants suggested the network norms played a larger role in their decisions about facility delivery than community norms.

Our finding that community norms are associated with facility delivery is supported by previous research. Specifically, Speizer and colleagues found that Ghanaian women’s perceptions that other husbands or MILs in their community supported facility delivery, and their perceptions of a higher number of women in their community who delivered in health facilities, were significantly related to facility delivery [11]. In the present study, the only community norm that remained significant in the adjusted model were women’s perceptions that women in their community delivered in a facility. Support from other men and other MILs in their community did not emerge as significant in the final model. While the support of MILs in influencing women’s place of childbirth has been well documented [28, 33], the present study suggests that perceptions related to where women within the community give birth were more powerful than perceptions of other MILs’, or other husbands’, support for facility delivery. Study findings suggest that community-level outreach that highlights the increasing rates of institutional delivery among rural Ghanaian women will be important to further encourage facility-based delivery.

Our examination of the relationship between personal network norms and facility delivery contributes to the sparce literature on the role of network norms on facility births. In prior qualitative research, network members, which include husbands, MILs, and mothers, seem to play prominent roles in influencing women’s pregnancy and delivery care experiences [28, 33, 34]. For example, women in rural Burkina Faso and rural Ghana relied on their husband and (mother-, father- and brother-) in-laws to make decisions about their use of facility-based pregnancy and delivery care [28, 34]. Resonating with the findings in the present study, the authors of these studies suggested that the influential role of network members included provision of needed resources. Our study, which uniquely quantifies the relationship between network norms and facility delivery, also revealed that network members provided women with critical support to enable their use of facility-based care. Our findings related to the importance of network norms and support from women’s personal network specifically underscores the need for continued efforts to promote support for facility-based pregnancy-related care among women’s personal networks. For example, health education during home visits, community gatherings, and prenatal care visits could further strengthen the role of network members in facilitating the use of facility delivery among rural Ghanaian women.

### Limitations

There are a few limitations worth noting in this study. The quantitative study was based on cross-sectional data; thus, we were unable to establish temporal relationships between network characteristics and use of facility delivery. As our analyses focused on women who had given birth three years prior to the survey administration, there is the potential for recall bias in women’s responses and the opinions of network members may have changed after women gave birth successfully at a health care facility. In cases where the gender of research assistants who administered surveys and conducted interviews did not match with study participants, response bias may have occurred. Participants were asked probing questions to address this potential bias. Also, the network data may be limited in that we did not directly survey or interview women’s personal networks. The qualitative interviews were conducted in native Ghanaian languages and transcribed and translated into English; errors in translations and nuances in meaning may have been missed. In anticipation of this concern, the research team maintained interview summaries and memos, and met regularly during the data collection process to debrief.

## Conclusion

Network and community norms are critical influences on women’s use of facility delivery. While we found the perception that most women in the community have facility delivery was associated with facility delivery, network norms were even more pronounced in women’s access and use of facility delivery during pregnancy. Our findings indicate that quality improvement initiatives are well positioned to impact both community and network norms in under-resourced communities like those in rural Ghana. These initiatives should focus on highlighting the shifting trend toward facility delivery in rural communities and the role of women’s personal networks in supporting facility-based pregnancy-related care.

## Data Availability

The datasets used and/or analyzed during the current study are available from the corresponding author on reasonable request.

## Abbreviations

IDIs: In-depth interviews
FGDs: Focus group discussions
MILs: Mothers-in-law
NR: Northern Region
CR: Central Region
